# Integrative network analysis suggests prioritised drugs for atopic dermatitis

**DOI:** 10.1186/s12967-024-04879-4

**Published:** 2024-01-16

**Authors:** Antonio Federico, Lena Möbus, Zeyad Al-Abdulraheem, Alisa Pavel, Vittorio Fortino, Giusy del Giudice, Harri Alenius, Nanna Fyhrquist, Dario Greco

**Affiliations:** 1 Finnish Hub for Development and Validation of Integrated Approaches (FHAIVE), Faculty of Medicine and Health Technology, Tampere University, 33100 Tampere, Finland; 2 Tampere Institute for Advanced Study, Tampere University, 33100 Tampere, Finland; 3 Institute of Biomedicine, University of Eastern Finland, Kuopio, Finland; 4 Faculty of Medicine, Human Microbiome Research Program, University of Helsinki, Helsinki, Finland; 5 Institute of Environmental Medicine (IMM), Karolinska Institutet, Stockholm, Sweden; 6 Division of Pharmaceutical Biosciences, Faculty of Pharmacy, University of Helsinki, 00100 Helsinki, Finland; 7 Institute of Biotechnology, University of Helsinki, 00100 Helsinki, Finland

**Keywords:** Atopic dermatitis, Network analysis, Disease module, Biomarkers, Drug discovery

## Abstract

**Background:**

Atopic dermatitis (AD) is a prevalent chronic inflammatory skin disease whose pathophysiology involves the interplay between genetic and environmental factors, ultimately leading to dysfunction of the epidermis. While several treatments are effective in symptom management, many existing therapies offer only temporary relief and often come with side effects. For this reason, the formulation of an effective therapeutic plan is challenging and there is a need for more effective and targeted treatments that address the root causes of the condition. Here, we hypothesise that modelling the complexity of the molecular buildup of the atopic dermatitis can be a concrete means to drive drug discovery.

**Methods:**

We preprocessed, harmonised and integrated publicly available transcriptomics datasets of lesional and non-lesional skin from AD patients. We inferred co-expression network models of both AD lesional and non-lesional skin and exploited their interactional properties by integrating them with a priori knowledge in order to extrapolate a robust AD disease module. Pharmacophore-based virtual screening was then utilised to build a tailored library of compounds potentially active for AD.

**Results:**

In this study, we identified a core disease module for AD, pinpointing known and unknown molecular determinants underlying the skin lesions. We identified skin- and immune-cell type signatures expressed by the disease module, and characterised the impaired cellular functions underlying the complex phenotype of atopic dermatitis. Therefore, by investigating the connectivity of genes belonging to the AD module, we prioritised novel putative biomarkers of the disease. Finally, we defined a tailored compound library by characterising the therapeutic potential of drugs targeting genes within the disease module to facilitate and tailor future drug discovery efforts towards novel pharmacological strategies for AD.

**Conclusions:**

Overall, our study reveals a core disease module providing unprecedented information about genetic, transcriptional and pharmacological relationships that foster drug discovery in atopic dermatitis.

**Supplementary Information:**

The online version contains supplementary material available at 10.1186/s12967-024-04879-4.

## Background

Atopic dermatitis (AD) is one of the most common chronic inflammatory skin diseases characterised by extended skin lesions leading to significant discomfort and reduced quality of life. Its pathophysiology arises from the interplay between genetic and environmental factors, leading to a wide range of symptoms, including skin inflammation, sensitisation, hyperkeratosis and pruritus. These manifestations are usually driven by an excessive T-cell activation and an increased production of inflammatory cytokines [1]. A definitive cure for AD does not exist and current treatments are aimed to ease the symptoms. The compounds employed in the clinical management of the disease are mainly represented by topical or systemic corticosteroids, which are known to have significant long-term side effects [2]. Other treatments include pimecrolimus and tacrolimus for patients that are non-responsive to simpler treatments while antihistamines are employed in case of severe itching. In this setting, there is a need for more effective and targeted treatments that address the root causes of AD. Therefore, identifying additional compounds targeting the molecular vulnerabilities of AD can help to define more effective therapies for patients.

De novo drug development is a long and expensive process [3] while the failure rate is above 90% including drug candidates in the preclinical stage [4]. While High-Throughput Screenings (HTS) represent the gold standard for modern drug development since they allow the parallel testing of thousands of compounds, they do not solve the problem of high costs in terms of time and resources. For these reasons, drug discovery is emerging as a viable solution to overcome these drawbacks [5]. At the same time, virtual screening is being increasingly introduced in the drug development routines, given its ability to reduce a priori the number of compounds selected for testing, hence improving the success rate. In a previous effort, we showed how the construction of virtual libraries of compounds can aid the computational assessment of thousands of compounds against the desired targets, reducing the number of compounds to undergo pre-clinical testing [5].

In recent years, in order to leverage the construction of tailored compound libraries, considerable efforts have been made to identify the cutaneous molecular alterations associated with AD. Advancements in omics technologies and the massive amounts of data currently available revealed a large number of deregulated genes and pathways in AD, allowing the discovery of novel biomarkers, disease subtypes/endotypes and therapeutic targets [6–9]. Manipulating and integrating these data into biologically meaningful results is still challenging especially in the light of the high disease and study heterogeneity. For these reasons, an exhaustive characterization of the pathogenetic mechanisms and therapeutic opportunities of AD have not been achieved yet [10, 11].

Network science is at the forefront to meet this challenge and its potential has been widely demonstrated [12, 13]. Network modelling of biomolecular processes enables the full integration and exploitation of heterogeneous (multi-)omics datasets, uncovering key molecular mechanisms responsible for the onset of several human diseases and facilitating the design of new and more effective treatments. Although several effective and specific treatments with tolerable side-effects are available for patients with AD [14], challenges such as the high disease heterogeneity as well as the effect of multimorbidity are demanding the development of further treatment solutions [15, 16]. To date, the potential of network science has not been yet exploited to facilitate drug discovery in AD.

**Fig. 1:**
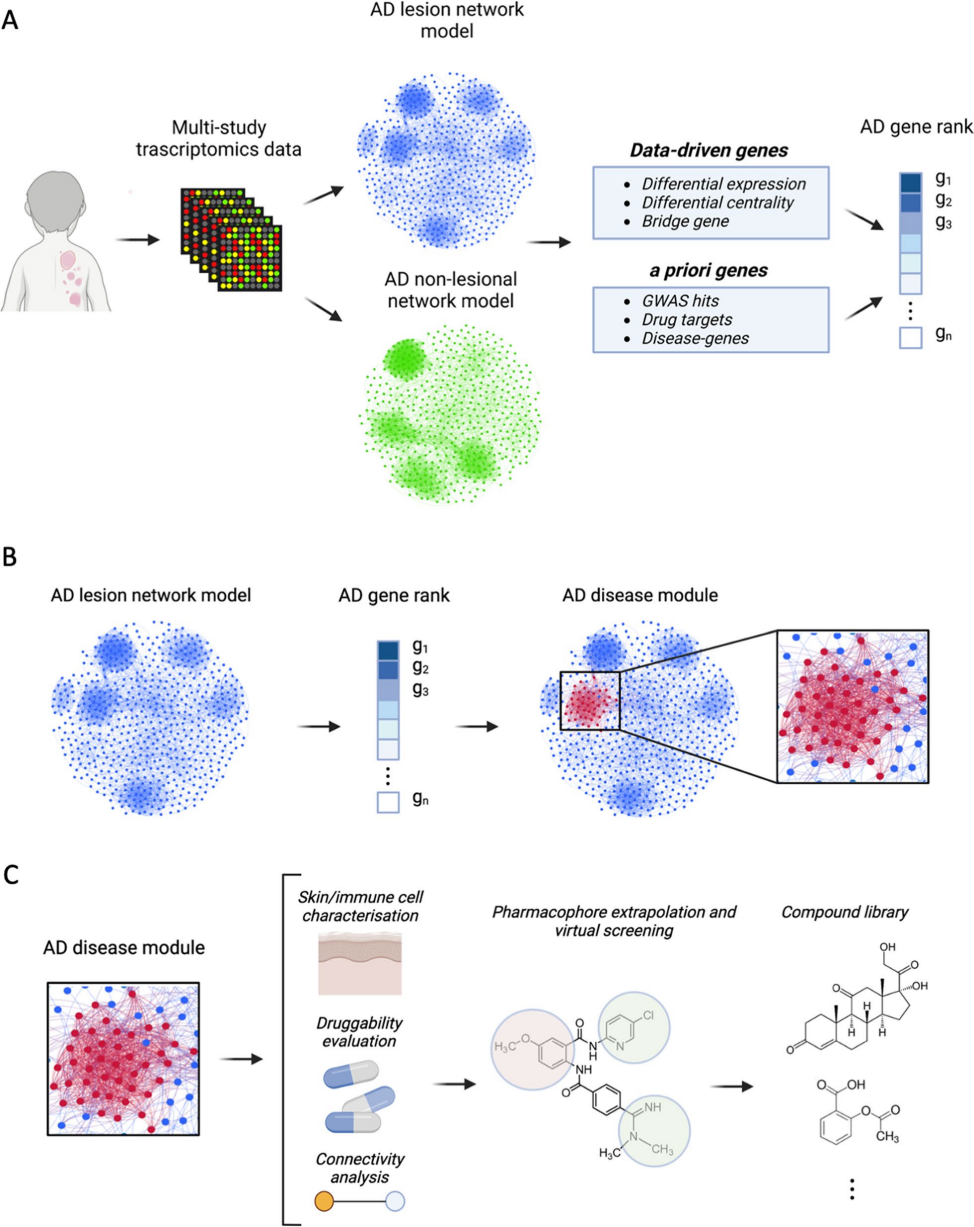
Overview of the analytical pipeline developed in this study. **A** Integration of publicly available transcriptomics datasets of lesional skin and non-lesional counterparts from atopic dermatitis patients. The data underwent meta-analysis and were utilised to infer co-expression network models for the lesional and non-lesional skin. Therefore, a gene rank was computed by exploiting information from data driven genes and genes with a priori knowledge of association with atopic dermatitis. **B** Identification of the atopic dermatitis disease module. **C** Characterisation of the disease module based on skin and immune cell transcriptional signatures, drug-target associations and intra-module connectivity. Druggability evaluation of the disease module, extrapolation of pharmacologically active pharmacophores and identification of novel compounds through virtual screening

In this study, we exploited network modelling to extrapolate a robust, cohesive and interpretable AD disease module, pinpointing dysregulated genes and processes underlying the eczematous lesions. We leveraged the potential of drugs targeting the AD module, via the identification of relevant pharmacophores and, through virtual screening, we prioritized drugs and drug targets to build a compound library to foster tailored and pharmacologically meaningful drug discovery predictions. Moreover, our investigations provide a vocabulary of chemical substructures to facilitate the development of novel compounds with improved therapeutic and pharmacokinetic properties and promote the shift from traditional drug development towards mechanistic drug design. The whole analytical pipeline implemented in this study is shown in Fig. 1.

## Methods

### Data collection and preprocessing

All the raw transcriptomics data collected and utilized in this manuscript are publicly available in the Gene Expression Omnibus (GEO) repository. The preprocessed data consist of 12 microarray-derived gene expression datasets of both lesional (337 samples) and non-lesional skin (542 samples) from patients with AD. GEO IDs of the collected datasets are reported in Table S1.

The preprocessing procedure was carried out as described in [17]. Differentially expressed genes for each dataset were identified through the use of the eUTOPIA software [18] by comparing the lesional skin samples with the non-lesional ones. For the analysis, eUTOPIA default parameters were used.

A priori information about AD were retrieved from several public repositories. GWAS hits were collected from the GWAS catalog (https://www.ebi.ac.uk/gwas/). Disease-gene associations and drug targets were retrieved from OpenTargets (https://www.opentargets.org/).

### Data scaling

All of the collected microarray datasets were combined for cross-platform normalization. In particular, the *pamr* R package (version *1.56.1*) [19] was used to mean-adjust the combined microarray data based on a batch variable representing the different datasets downloaded from GEO.

### Network inference and analysis

Two distinct co-expression networks were inferred by using the gene expression profiles of the lesional and non-lesional skin samples from all the included studies and selecting the genes common to all the platforms.The co-expression networks were inferred as already described in [20] through the use of the *INfORM* algorithm [21]. We set up *INfORM* with the same parameters used in [20]. We used the clr [22], aracne [23] and mrnet [24] algorithms with the following correlation and mutual information measures: Pearson correlation, Kendall correlation, Spearman correlation, empirical mutual information, Miller-Madow asymptotic bias corrected empirical estimator, Schurmann-Grassberger estimate of the entropy of a Dirichlet probability distribution and a shrinkage estimate of the entropy of a Dirichlet probability distribution, as implemented in the *minet* Bioconductor package [25]. In order to carry out a network community detection we used the walktrap algorithm [26], implemented in *INfORM*. All computations performed on the inferred networks were carried out through the use of the *igraph* Bioconductor package [27]. The rendering of co-expression networks was performed by employing the *gephi* software [28]. In this manuscript, we show a reduced representation of the networks (subgraph) in order to facilitate the visualisation.

### AD gene rank construction

The ranked compendium constructed in this study encompasses genes whose association with AD was inferred in two ways: (1) data driven association, resulting from the meta-analysis of the collected transcriptomic datasets, and the analysis of network connectivity, including differential centrality analysis and the analysis of the bridge genes; (2) association deriving from a priori knowledge, including genomic variants associated with AD, annotated disease-gene associations and drug-target associations.

#### Genes deriving from data-driven association

1. Gene expression meta-analysis: Meta-analysis of the transcriptomic datasets was implemented as described in del Giudice et al. [29] according to a consensus strategy previously reported to be suitable for gene signature retrieval from omics data [30, 31].
  - Briefly, the pipeline is based on the combination of effect-size, p-value based and rank-product methods. Fisher test is the standard meta-analysis approach to combine p-values. Effect-size strategies are considered the gold standard to assess within- and between-study variation, while accounting for small sample sizes. Finally, the Rank-product method allows combining individual studies according to the within-study gene expression analysis. The intermediate individual ranks were obtained by using the “sumlog” function of the *metap* R package (Dewey, M. metap: meta-analysis of significance values. CRAN (2022)), the “effect_sizes” function of the *esc* R package (Lüdecke, D. Esc: Effect Size Computation for Meta Analysis. CRAN (2019)), and the “RP-advance” function of the *RankProd* R package [32], respectively. The final consensus gene rank was generated through the Borda function of the *TopKlists* R package [33].
2. Differential centrality analysis: We performed differential centrality analysis as already described in [20]. In detail, for each of the networks, their node betweenness, closeness and degree centralities were calculated with the Python’s *NetworkX* package (Python 3.6, NetworkX 2.3). The nodes were ranked according to each of the centrality measures. For each network, the median rank of the nodes based on the rankings of the three centrality measures were calculated. To compare the network of the lesional skin with the non-lesional one, the absolute difference between the median ranks of the two networks was calculated and the genes were ranked accordingly.
3. Bridge gene analysis: The identification of the bridge genes was conducted as reported in [20]. In our previous study, we hypothesized that, by studying the connectivity patterns among known disease genes, it is possible to identify additional associated genes. Therefore, in this study, we identified all the genes connecting pairs of known AD-associated genes within each of the networks (lesional and non-lesional, respectively), and hence acting as a bridge.

#### Genes deriving from a priori knowledge association

1. Genes mapped to genomic variants associated with AD were retrieved from the GWAS catalog [34], [https://www.ebi.ac.uk/gwas/].
2. AD disease genes were retrieved from the DisGeNet repository [35].
3. Drug-target associations, retrieved from the OpenTarget database [36].

The compendium was ultimately built by merging genes from both categories and ranking them on the basis of the total number of evidence of association with AD.

### Inference of the AD disease module

The AD disease module was inferred by using the DIAMOnD algorithm [37]. DIAMOnD works under the hypothesis that disease modules are not usually represented by highly cohesive subgraphs, differently from functional modules, which often overlap with topological communities. DIAMOnD identifies disease modules by making use of a set of genes that are already known to be associated with the disease of interest. The algorithm assesses whether a certain gene holds more connections to seed genes than expected, calculating a connectivity* p*-value. In our study, the genes of the compendium holding at least 3 pieces of evidence of association with AD were used as seed genes for the algorithm in both the lesional and the non-lesional network models. Genes that were identified by the DIAMOnD algorithm showing a p-value ≤ 0.05 in each network were considered for analysis. We therefore selected genes that were significant in the lesional network but not in the non-lesional one. The AD disease module was eventually defined by merging such a set of genes with the seed genes.

### Skin and immune cell type specific gene enrichment

The enrichment of skin and immune cell type-specific genes in the disease module has been evaluated by utilizing the single-cell RNA-Sequencing derived signatures publicly available at the Human Protein Atlas (https://www.proteinatlas.org/). The skin cell types included in this analysis are the following: adipocytes, eccrine cells, endothelial cells, fibroblasts, hair, keratinocytes, Langerhans cells, macrophages, mast cells, melanocytes, mitotic, outer, plasma cells, sebaceous cells, smooth cells, skin T-cells. The selected pool of immune cell types comprise: T-cells, monocytes, macrophages, granulocytes, B-cells, basal cells, dendritic cells, erythroid cells, Langherans cells, NK-cells, plasma and suprabasal cells.

One tail gene set enrichment analyses (GSEA) were performed through Kolmogorov–Smirnov statistics, as implemented in the *stats* R package. Overrepresentation tests were performed by using the *bc3net* CRAN package [38].

### AD disease module druggability evaluation

To evaluate the druggability of the AD disease module, we combined drug-target, disease-medication and disease-disease similarity information retrieved from DrugBank [39], STITCH [40], Open Targets [41], Pharos [42], and the GWAS catalog [34]. To integrate disease data from different data sources we annotated their disease names with NCBI MedGen concept IDs as a common vocabulary.

Similarity of AD with other diseases was computed based on genes encompassing genetic variants associated with the disease as annotated in the GWAS catalog. We merged phenotypes representing AD in the GWAS catalog (eczema and atopic dermatitis) by combining their associated genes. Diseases with fewer than 5 genes associated were removed from the analysis. Using the gene lists of 611 diseases, we calculated pairwise disease similarities by computing the Jaccard index, cosine similarity, Sørensen–Dice coefficient, and overlap coefficient, as well as disease distances by the Euclidean and Hamming distance. Similarity metrics were inverted to measures of distance by subtraction from 1. Euclidean and Hamming distances were scaled to a 0–1 range by division by the maximum. We computed the Ipsen-Mikhailov distance among the six distance measures using the netdist function from the R nettools package (https://rdrr.io/cran/nettools/). A consensus distance was computed from the six distance measures by taking the mean in a hierarchical fashion based on hierarchical clustering of Ipsen-Mikhailov distances. Based on the consensus distances, the diseases were ranked from the most similar to the least similar to AD.

Subsequently, we extracted disease-drug-target associations from DrugBank, STITCH, Open Targets and Pharos and kept only the targets *i.e.*, genes belonging to the AD disease module. We prioritised drug candidates based on their therapeutic application for diseases similar to AD.

Virtual screening was performed on an in-house library containing chemical compounds from Drugbank, Pharos and Open Targets.

### 2D and 3D structure representation for the investigated compounds

The 2D sketcher tool of Maestro molecular modeling suite was used to build the 2D structures from the smiles of the compounds.

The LigPrep module of the Maestro molecular modeling suite was used for ligand preparation. Neutral pH was used in the ligand preparation with OPLS4 force field and determining chiralities from 3D structure options were selected [43, 44].

### Pharmacophore identification and virtual screening

The Phase tool of Maestro was used to develop a common pharmacophore hypothesis for the active ligands and to use it for pharmacophore-based virtual screening. The virtual screening was performed on an in-house library containing chemical compounds from Drugbank, Pharos and Open Targets to identify compounds that share the selected pharmacophore and features [45].

### ADME properties calculation

To identify the ADME (Absorption, Distribution, Metabolism, Excretion) properties for the ligands, the QikProp tool in Maestro was used. (QikProp, Schrödinger, LLC, New York, NY, 2021).

## Results

### An AD-specific gene module represents relevant functions within the lesional network model

Complex diseases, including atopic dermatitis, often result from the impairment of complex cascades of interactions between molecular effectors. In this context, the construction of disease network models has been shown to be a valid method in order to disentangle such complex interactions, and identify key players in the pathophenotype of diseases [37]. Here, we inferred two transcriptome-wide gene co-expression networks modelling both the lesional and the non-lesional skin molecular buildup of AD patients, respectively. Since the networks were inferred from all the genes common to all the collected transcriptomics datasets, both networks were composed of 17,903 nodes, while the number of edges was 1,649,569 and 1,924,001 for the lesional and the non-lesional, respectively.

Next, we exploited such networks to define a disease module for AD in order to identify a core set of genes that might play a role in the disease and might function as therapeutic targets. To do so, we compiled a ranked compendium of genes whose association with AD is based on (1) a priori knowledge about their involvement in AD and (2) data-driven analyses of the transcriptomics datasets and co-expression networks (see Methods). Subsequently, we selected 1234 genes (here named “seed genes”) having at least three supporting evidence of their association with AD as reference genes for the identification of the AD disease module. We carried out this analysis on both the lesional and the non-lesional network by utilising the same set of seed genes. We therefore identified 1218 genes that are significantly more connected with the seed genes in the lesional network but not in the non-lesional one. In this way, we defined the atopic dermatitis module, encompassing 2452 genes.

**Fig. 2:**
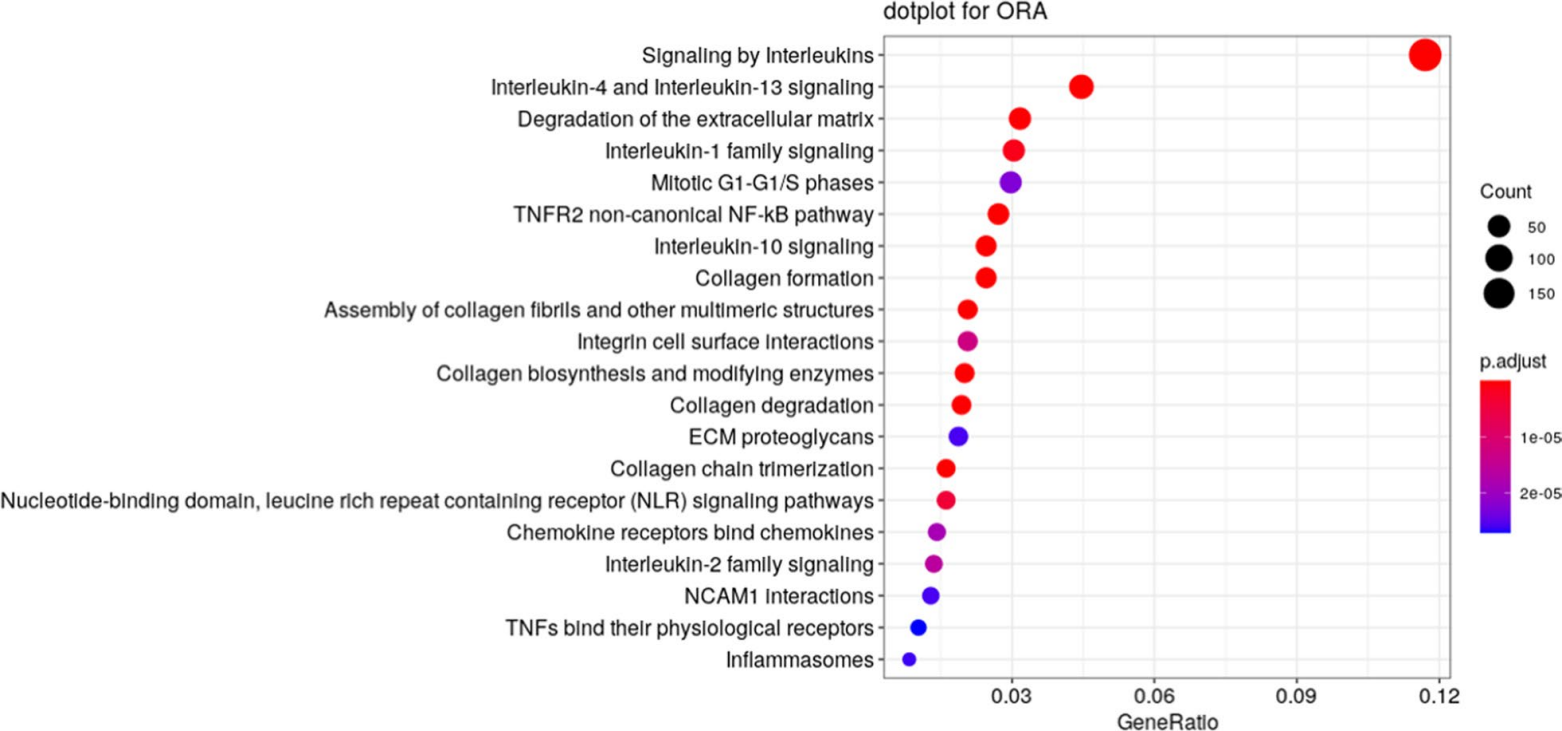
Pathways enriched by genes belonging to the AD disease module

We characterised the functional processes represented by such genes by performing an overrepresentation analysis by Fisher’s exact test (Fig. 2).

Interestingly, 181 genes of the disease module are annotated in the “*Signaling by Interleukins*” pathway, which is the most overrepresented in our analysis (*adj. p-value* = 2.78e-37). Similarly, other pathways related to interleukin signalling and extracellular matrix remodelling were found to be overrepresented, including “*Interleukin-4 and Interleukin-13 signaling” (adj. p-value* = 3.97e-29*)*, “*Degradation of the extracellular matrix” (adj. p-value* = 1.45e-07*)*, “*Interleukin-1 family signaling” (adj. p-value* = 1.33e-06*)*, “*TNFR2 non-canonical NF-kB pathway” (adj. p-value* = 1.3e-08*)*, “*Interleukin-10 signaling” (adj. p-value* = 1.78e-21*)*, “*collagen formation” (adj. p-value* = 3.18e-08*)*, “*collagen degradation” (adj. p-value* = 9.8e-08*)*, among others.

**Fig. 3:**
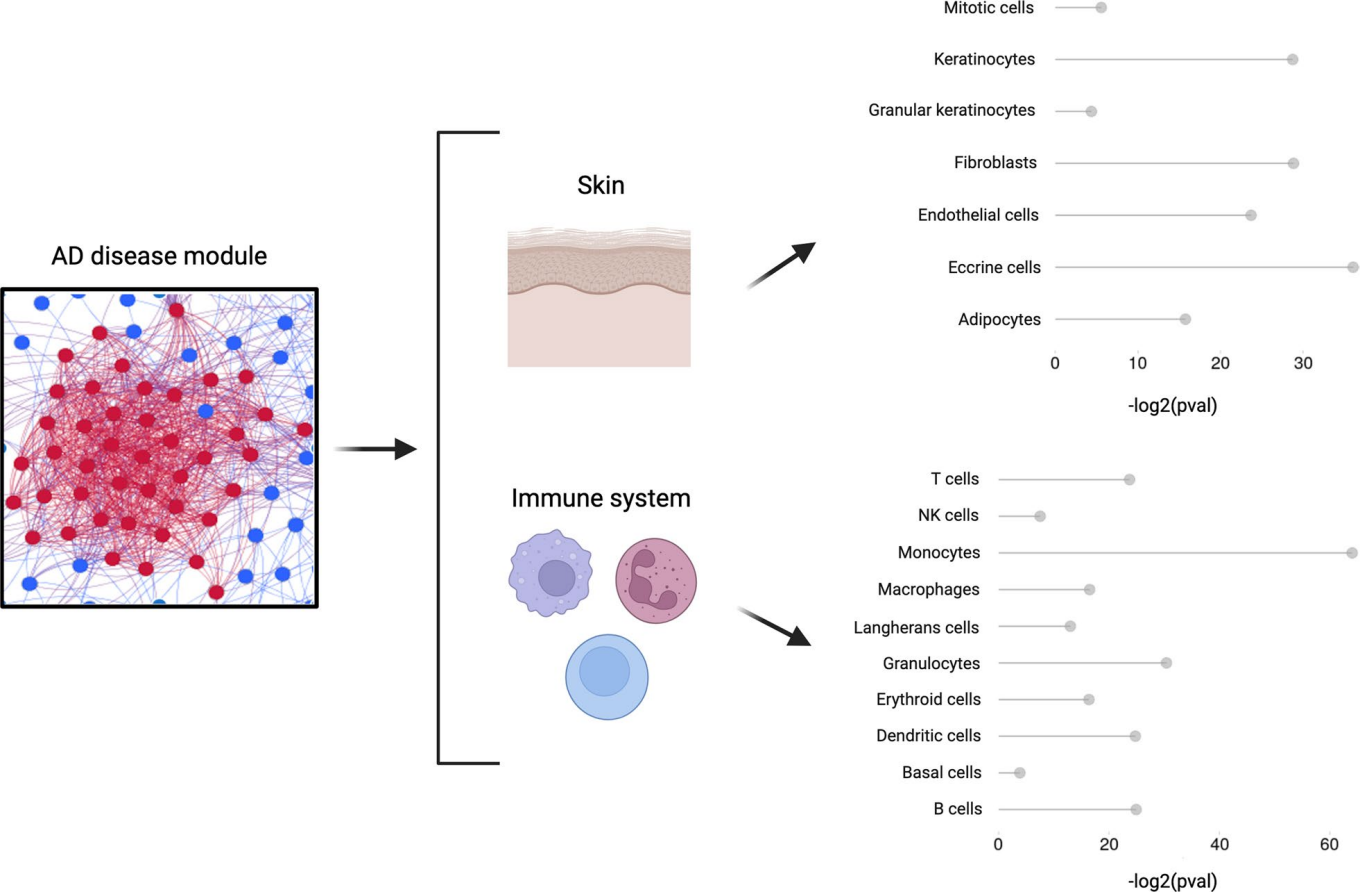
Results of the enrichment analysis of skin and immune cell type-specific genes within the AD disease module

The manifestation of AD lesions arises from a dysregulated cutaneous immune response and an impairment of the skin barrier, which is normally composed by a wide range of cell types contributing to its homeostasis. AD lesional skin is characterised by a chronic inflammatory milieu due to several cellular dysfunctions including a disturbed epidermal barrier integrity, increased immune cell infiltration, abnormal secretion from sebaceous cells and eccrine gland cells. Therefore, we further characterised the genes within the AD module, by considering the known patterns of expression in multiple skin and immune cell types. Our analysis supports the functional annotation reported above and identifies cell types that underlie relevant functions. We, then, exploited publicly available gene expression signatures from several skin cell types from the Human Protein Atlas database by performing a Gene Set Enrichment Analysis (GSEA) to assess the enrichment of cell type-specific genes within the AD disease module (Fig. 3). Our analysis highlighted a significant enrichment of genes specifically expressed in keratinocytes (different stages of differentiation, *adj. p-value* = 1.64e-12), eccrine gland cells (*adj. p-value* = 3.29e-15), skin fibroblasts (*adj. p-value* = 1.64e-12), skin endothelial cells (*adj. p-value* = 1.91e-10), skin adipocytes (*adj. p-value* = 4.30e-07), skin mitotic cells (*adj. p-value* = 9.56e-03), and granular keratinocytes (*adj. p-value* = 2.72e-02). Similarly, since the dysregulation of immune cells and their crosstalk plays a crucial role in the development and maintenance of atopic dermatitis, we also assessed the presence of cell signatures related to the immune compartment within the AD disease module. As a result, we obtained a significant enrichment for the following immune cells: macrophages (*adj. p-value* = 1.04e-21), monocytes (*adj. p-value* = 3.2e-25), T-cells (*adj. p-value* = 1.04e-21), granulocytes (*adj. p-value* = 5.2e-19), Langerhans cells (*adj. p-value* = 7.89e-13), dendritic cells (*adj. p-value* = 6.71e-14), B-cells (*adj. p-value* = 2.23e-14), NK-cells (*adj. p-value* = 2.69e-09). The entire list of skin and immune cell types included in this analysis is described in the Methods section.

### Co-expression patterns underlying AD lesional skin drives biomarker discovery

We here propose that investigating connectivity patterns within the disease module could serve as an alternative and effective method for biomarker prioritisation. Traditionally, biomarkers have been understood as disrupted expression profiles of single genes occurring in skin lesions. However, network medicine suggests that specific connectivity patterns involving interactions between two or more genes could be utilized as biomarkers for these lesions.

Therefore, to fully exploit the informative power of the AD disease module, we characterised the edges (representing co-expression relationships between two genes) connecting genes within the module.

**Fig. 4:**
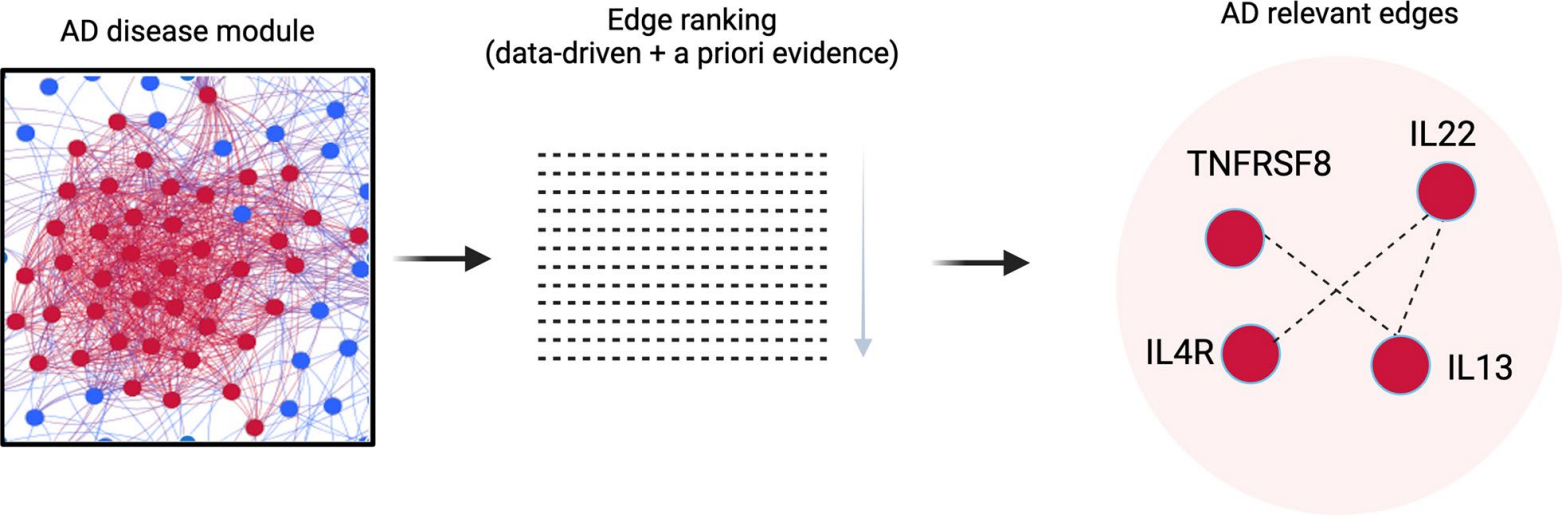
Selection process to retrieve AD-relevant edges

We evaluated the relevance of each gene within the AD module by collecting multiple data-driven aspects (transcriptome meta-analysis, differential centrality, and bridge gene analysis) as well as prior-knowledge (GWAS, drug targets, disease genes) (Fig. 1A). We used data-driven and prior evidence to rank the edges connecting genes within the module (Fig. 4).

Based on these criteria, *TNFRSF8–IL13*, *IL22–IL13* and *IL4R–IL22* are the top 3 ranked edges suggesting the high relevance of these genes and their co-expression for AD. The top 100 edges connecting relevant genes are reported in Additional file 2. Next, we divided the edges in two sets: the first group includes edges connecting pairs of seed genes (used to infer the AD disease module), while in the second, edges for which only one of the genes is a seed gene were included. In this way, we characterised functional processes involving only seed genes, and non-seed genes within the disease module via direct connection to seed genes. The set of edges connecting seed genes with each other enriched several *signalling by interleukins* pathways, *extracellular matrix organisation and degradation*, and cell cycle related pathways, such as *mitotic G1/S transition* and several pathways related with collagen deposition and modification, such as *Collagen formation*, *collagen degradation*, *collagen biosynthesis and modifying enzymes*, *collagen chain trimerization.* On the other hand, the set of edges connecting seed genes with other genes enriched *Processing of capped intron-containing pre-mRNA*, *mRNA splicin*g pathways and *Rho-GTPase cycle*. This analysis allows us to enrich the information contained in the module by interactional properties of data-driven and prior knowledge-driven genes contained into it.

### Definition of a custom library of compounds for drug discovery in AD

**Fig. 5:**
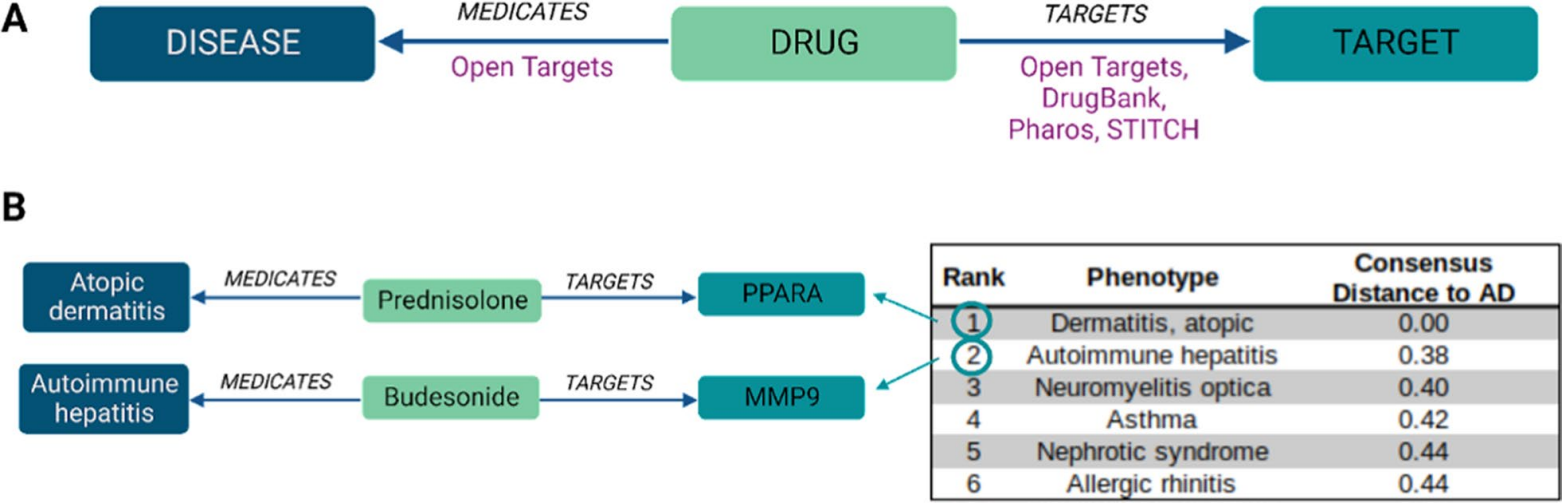
Prioritisation of drug targets

Subsequently, we constructed a custom library of compounds to foster drug discovery for the definition of efficient therapeutic compounds. We extracted drug-diseases and drug-target relationships from public repositories, including DrugBank [39], STITCH [40], Open Targets [41], Pharos [42]. Notably, we retrieved 285 drugs targeting 635 genes of the AD disease module. To provide further relevance to the drugs within the module, we prioritised compounds that are used to treat diseases that share a similar genetic background with AD. Therefore, we computed a disease-disease similarity and prioritised diseases showing the highest similarity with AD based on genes with genomic variants. We extracted drug-disease relationships and obtained a list of disease-drug-target relationships (Fig. 5A) by merging them with the drug-target relationships. We ranked the list based on the AD-similarity of the disease in the disease-drug-target triplets including AD itself (*i.e.*, drug-target entities linked to AD would always have the highest rank). Afterwards, we filtered such a rank on the drug target genes belonging to the AD disease module (Fig. 5B).

The 285 drugs included in the rank are employed in the treatment of 66 diseases. The most similar disease to AD was autoimmune hepatitis, followed by neuromyelitis optica, asthma, nephrotic syndrome and allergic rhinitis. Drugs used to treat AD such as prednisolone, cyclosporine, tacrolimus, and dupilumab ranked at the top. Moreover, drugs used to treat diseases similar to AD ranked at lower positions. Abatacept and mycophenolate mofetil are used to treat autoimmune hepatitis, the disease with the highest similarity to AD based on genetic susceptibility (Additional file 3). We here hypothesised that potentially effective drugs for AD share relevant structural properties, namely pharmacophores.

Therefore, we extrapolated relevant pharmacophores of such drugs in order to identify the minimal active substructure of such compounds (Additional file 1: Table S2).

In order to extrapolate more accurate pharmacophores, we selected 6 features responsible for desired effects.

Among all the drugs included in the disease-drug-target rank computed in the previous step, 19 share the highest ranked pharmacophore hypothesis including prednisolone, tacrolimus, and pimecrolimus, which are approved for treatment of AD. We further tested the hypothesis that additional drugs, not initially included in our list, could be proposed for AD treatment if relevant pharmacophores are included in their molecular structure. To this end, we performed a virtual screening, which consists in screening large libraries of compounds in search of specific structural properties of interest.

As a result, we identified 1,505 compounds that share the selected pharmacophore and other relevant structural features (Additional file 4). Corticosteroids are the most represented class of drugs at the top of the rank, such as fluocinonide and halopredone acetate. NCX1022, a NO-releasing derivative of hydrocortisone, placed at the 3rd position of the rank, has been investigated in a murine model of AD and showed anti-inflammatory effects [46]. Given the relevance of this investigational compound in the library, we assessed whether such a compound shares molecular targets with drugs that are approved for inflammation management. Interestingly, our prediction shows that NCX1022 shares 21 molecular targets with abrocitinib and upadacitinib.

Desoximetasone, which is a glucocorticoid used for the treatment of inflammatory conditions, skin allergies and dermatoses, also ranked 12th among the drugs.

We finally computed the Adsorption, Distribution, Metabolism and Excretion (ADME) values of the identified library of compounds (Additional file 5, Additional file 1: Figure S1).

​​The ADME analysis reveals that NCX1022 shows higher metabolism rate compared with the other drugs. Moreover, NCX1022 shows medium oral absorption, while the other drugs from the top 10 showed high oral absorption. These findings show that the top 10 ranked drugs have medium to high ADME values, which is a predictive factor for the bioavailability and for the maximum therapeutic efficacy.

## Discussion

Network models provide a powerful framework for integrating heterogeneous datasets and are at the forefront of multi-omics data analysis and interpretation, enabling the discovery of hidden patterns, relationships, and insights that would otherwise be difficult to detect using traditional approaches. By leveraging the interconnectedness and interdependencies within a co-expression network, such as nodes representing genes and edges representing relationships between them, network models offer a holistic view of complex systems, such as human diseases.

In this study, we provide a network-based modelling approach integrating the biggest number of cutaneous transcriptomic datasets of AD, to date. We computed gene co-expression networks of lesional and non-lesional skin, both consisting of 17,903 genes. Notably, the lesional network contained substantially fewer edges (1,649,569) than the non-lesional network (1,924,001) highlighting disrupted patterns of co-expression in AD skin lesions and indicating dysfunctional gene regulatory mechanisms.

In recent years, the advancement of analysis strategies of disease-related networks, led to the identification of so-called “disease modules”. Disease modules are subsets of genes highly interconnected within a disease network from which we can gain insights into the pathophysiology of the disease under study. Genes belonging to a disease module often work together in specific pathways or functional units, shedding light on how impaired molecular mechanisms contribute to the disease development or progression [47, 48]. By combining data-driven evidence with prior knowledge, we here identified a robust disease module of the AD skin lesion encompassing 2452 genes.

As this community of genes related to the atopic lesion was derived by several features including gene deregulation, gene–gene co-expression relationships, and genetic variation associated with the disease, the computation of the disease module overperforms the computation of differentially expressed genes as usually identified and used for downstream analysis in single-dataset transcriptomic studies [37].

While gene sets derived from the analysis of differential expression often reveal a variety of pathways related to inflammation and innate immune responses, the disease module identified in this study exhibits the most significant enrichment for pathways targeted by state-of-the-art therapeutic solutions for AD, such as biologics targeting IL4-IL13 signalling (dupilumab and tralokinumab), and drugs targeting genes such as *JAK1*, *JAK2*, *JAK3* and *TYK2*, which are pivotal actors in the Jak-Stat signalling pathway (barticitinib, upadacitinib and abrocitinib).

However, the lack of efficacy in some patients highlights the need for other effective molecules, and underlines the importance of developing targeted medicine tailored to patients characteristics. Further enriched pathways reveal the relevance of TNF, NF-kB, and chemokine signalling as well as a strong signature of epidermal barrier dysfunction. Here, we also identified a group of genes that is overrepresented in a high number of known dysregulated pathways in the AD lesion. Such genes, including MMP9, MMP3 and COL1A2 among others, might represent key players in the molecular impairment underlying the aberrant immune response and the dysfunctional barrier as characteristic for AD. In fact, keratinocytes express MMP9 as an important factor in the maintenance of the epithelial barrier function [49].

The analysis of the disease module revealed transcriptomic signatures from abundant cell types in the skin such as keratinocytes, eccrine gland cells, fibroblasts, and endothelial cells as well as immune cells such as lymphocytes and innate immune cells indicating that a complex dysregulated multi-cellular system sustains the phenotype under study. Understanding such complex multi-cellular interactions is crucial for advancing disease modelling and therapeutic interventions. In this perspective, it emerges the need for more sophisticated disease modelling procedures that incorporate the interactions and crosstalk among different cell types, which can lead to more precise and effective treatments.

Importantly, the characterisation of the gene connectivity within the disease module leveraged fundamental insights in the gene deregulation underlying the skin lesion. In fact, while relationships between known AD-associated genes are overrepresented in pathways that are typically disrupted in immune-mediated dermatological diseases, such as interleukin signalling, extracellular matrix organisation, collagen formation and degradation, connection between AD-associated and non-associated genes underlined mRNA processing indicating that the gene regulation machinery is tightly intertwined with known AD biomarkers such as interleukins and chemokines to sustain the AD pathological phenotype.

In this study, we investigated the interactional properties of genes belonging to the disease module, so as to give further insights into its functional characterisation. We demonstrated that, ranking the edges based on data-driven and prior knowledge-driven evidence of association with AD, is a valid approach to fully exploit network medicine principles to prioritise putative biomarkers of disease.

In fact, we hypothesise that exploiting properties of connectivity patterns within the disease network model can be an alternative and efficient tool for biomarker identification. The paradigm is that, while traditionally a biomarker is intended as a disrupted expression profile of a single gene specifically occurring in the skin lesion, network medicine suggests that certain patterns of connectivity involving two or more interacting genes could be exploited as biomarkers of lesions.

While the use of precompiled drug libraries is the state of the art in drug discovery, the principles by which the drugs are included are usually not evaluated. However, a rational inclusion process of drugs in libraries for drug discovery would enable a more effective process with a sensible improvement of the predictions, as we recently demonstrated in a case study on COVID-19 drug repositioning [5].

Since disease modules provide a formal perspective over the complexity of disease mechanisms, network pharmacology approaches can take advantage of this knowledge by considering the dependencies among genes belonging to the disease module. Given the high reliability of disease modules identified by integrating data-driven and a priori knowledge about the disease under study, compound libraries can be, in turn, designed in order to target multiple components belonging to the disease module, aiming to optimize the identification of compounds putatively effective for the disease. The definition of tailored compound libraries based on disease modules can leverage the identification of compounds that can have a broader impact on the disease phenotype and offer potential synergistic effects. Although the employment of network pharmacology approaches to identify candidate drugs and targets is well established, the possibility of using such principles to suggest desired structural properties to be considered in the discovery of drug candidates is less explored. Indeed, while current network pharmacology approaches are centered on molecular biology aspects, here we establish the paradigm that intimately integrates molecular biology with computational chemistry in order to construct tailored compound libraries.

We here demonstrate how the disease module can assist in building a custom compound library for drug discovery in AD. To do so, we prioritised common pharmacophores of drugs employed in the treatment of AD or other similar phenotypes whose target genes belong to the disease module. We identified a substructure shared among the investigated drugs. Afterwards, we performed a virtual screening on multiple high-dimensional repositories and defined a custom drug library to tailor drug discovery efforts towards the treatment of atopic dermatitis. Our results show that, although drugs that are employed in the treatment of AD dominate the ranked library, other categories of drugs, employed in diseases other than AD, are included in the rank. This aspect gives value to our data-driven approach for the construction of custom compound libraries and validates the ability to identify putative novel compounds that can be taken into consideration in future drug discovery predictions. On the other hand, our results do not mean to give any therapeutic indication for atopic dermatitis patients.

In this study, we built upon our previous manuscript focused on network analysis of transcriptomics data from lesional and non-lesional skin of psoriasis patients. In the present study we report the following advancements: (1) we implemented an ensemble meta-analysis method to extract robust gene expression signatures from transcriptomics data, which includes based on the combination of effect-size, p-value based and rank-product methods; (2) we identified an AD disease module by investigating the pattern of connectivity of AD-associated genes to enlarge the set of known AD biomarkers and focus the pharmacological investigations on novel putative drug targets; (3) we performed a more extensive druggability evaluation compared to our previous manuscript. In particular, we enlarged the set of drugs employed for AD treatment by prioritising drugs that are employed in the treatment of diseases that have a similar genetic background to AD in terms of genomic variants. Therefore, we compiled a compound library by carrying out a pharmacophore analysis to include drugs that encompass relevant structural units in their molecular structure, and that therefore could be used to focus future drug discovery efforts; (4) we envisioned that investigating connectivity patterns within the disease module could serve as an alternative and effective method for biomarker prioritization. In fact, we hypothesise that disease biomarkers could be represented by gene–gene interactions, sustained by multi-layer evidence of data-driven and a priori relevance, rather than the expression deregulation of single genes; (5) we extend the search in drug discovery by providing a vocabulary of chemical substructures that have the potential to mediate specific therapeutic effects on certain regions of the network and facilitating a shift from traditional drug development towards mechanistic drug design.

This study suffers from certain limitations due to the limited amount of clinical data available along with the transcriptional profiles obtained from public repositories. The absence of detailed clinical information prevents us from establishing meaningful associations between the disease module and relevant clinical parameters, such as disease severity and elapsed time since initial diagnosis. Furthermore, the lack of information on whether patients underwent active pharmacological therapy precludes us from discerning any potential influence of ongoing or terminated pharmacological treatments on the druggability profile of the disease module, which resulted, eventually, in the tailored compound library.

## Conclusions

In conclusion, our investigation significantly enhances our understanding of the molecular basis of AD by providing novel and unprecedented insights into the mechanistic and pharmacological relationships driving the AD phenotype. These results hold promise in identifying novel molecular targets and drug candidates, thereby propelling drug discovery research towards the development of effective therapeutic interventions for AD.

### Supplementary Information

**Additional file 1. Table S1.** The table reports the datasets and the number of lesional and non lesional samples from AD patients included in this study. **Table S2.** The table shows the pharmacophore hypotheses as well as their Phase hyposcores beside the BEDROC (Binary Ensemble Differential Relaxation Organizer) metric, which is used to evaluate the performance of pharmacophores and the EF1 (Enrichment Factor 1%), which is a statistical metric commonly used in virtual screening and drug discovery to evaluate the ability of a computational method to identify the most promising hits from a large database of compounds. The hypothesis that ranked highest based on such criteria was AAAHHH_2. **Figure S1.** Physicochemical properties summarising ADME properties and 2D structures of the top ranked drugs resulting from the virtual screening. The red area represents ideal ADME values for pharmaceutical drugs. The ADME analysis reveals that NCX1022 shows higher metabolism rate compared with the other drugs. Moreover, NCX1022 shows medium oral absorption, while the other drugs from the top 10 showed high oral absorption. These findings show that the top 10 ranked drugs have medium to high ADME values, which is a good predictive factor for the bioavailability and for the maximum therapeutic efficacy.

**Additional file 2.** The file indicates the top 100 edges of the AD disease module ranked by the relevance of the interacting genes.

**Additional file 3.** The file reports drugs used to treat diseases similar to AD targeting genes within the disease module. The file reports diseases, drugs, target genes and source of the association.

**Additional file 4.** Drugs sharing the selected pharmacophore and other relevant structural features resulting from the virtual screening.

**Additional file 5.** Adsorption, Distribution, Metabolism and Excretion (ADME) values of drugs identified through virtual screening.

## Data Availability

Data utilised in this study are publicly available in Zenodo at the following URL: https://zenodo.org/records/4009497.

## Abbreviations

AD: Atopic Dermatitis
ADME: Absorption, Distribution, Metabolism, Excretion
COVID-19: Coronavirus Disease 2019
GEO: Gene Expression Omnibus
GSEA: Gene Set Enrichment Analysis
GWAS: Genome Wide Association Study
IL4: Interleukin 4
IL4R: Interleukin 4 Receptor
IL13: Interleukin 13
IL22: Interleukin 22
NF-kB: Nuclear Factor Kappa B
NO: Nitric Oxide
OPLS4: Optimised potential for liquid simulations
TNF: Tumor Necrosis Factor
TNFRSF8: Tumor Necrosis Factor Receptor Superfamily 8
