# Supplementary material for: Integrative network analysis suggests prioritised drugs for atopic dermatitis
Source: J Transl Med. 2024 Jan 16;22:64. doi: 10.1186/s12967-024-04879-4 (PMC10792836; doi:10.1186/s12967-024-04879-4)
Supplement: Supplementary file 1 — Additional file 1. Table S1. The table reports the datasets and the number of lesional and non lesional samples from AD patients included in this study. Table S2. The table shows the pharmacophore hypotheses as well as their Phase hyposcores beside the BEDROC (Binary Ensemble Differential Relaxation Organizer) metric, which is used to evaluate the performance of pharmacophores and the EF1 (Enrichment Factor 1%), which is a statistical metric commonly used in virtual screening and drug discovery to evaluate the ability of a computational method to identify the most promising hits from a large database of compounds. The hypothesis that ranked highest based on such criteria was AAAHHH_2. Figure S1. Physicochemical properties summarising ADME properties and 2D structures of the top ranked drugs resulting from the virtual screening. The red area represents ideal ADME values for pharmaceutical drugs. The ADME analysis reveals that NCX1022 shows higher metabolism rate compared with the other drugs. Moreover, NCX1022 shows medium oral absorption, while the other drugs from the top 10 showed high oral absorption. These findings show that the top 10 ranked drugs have medium to high ADME values, which is a good predictive factor for the bioavailability and for the maximum therapeutic efficacy. [file 12967_2024_4879_MOESM1_ESM.pdf]

GEO ID	Publication	# AD lesional samples	# AD non-lesional samples
GSE107361	PMID: 29731129	37	66
GSE120721	PMID: 35784319	15	15
GSE120899	PMID: 30528828	18	18
GSE130588	PMID: 30194992	50	41
GSE27887	PMID: 21762976	9	8
GSE32924	PMID: 21388663	13	12
GSE36842	PMID: 22951056	16	8
GSE5667	PMID: 20625511	83	294
GSE58558	PMID: 24786238	18	16
GSE59294	PMID: 25482871	15	8
GSE95759	PMID: 28977706	4	3
GSE99802	PMID: 30121291	59	53

Table S1 - The table reports the datasets and the number of lesional and non lesional samples from AD patients included in this study.

Hypothesis	Phase Hypo Score	EF1%	BEDROCK	ROC	AUAC	Matches
AAAHHH_2	0.63	5.3	0.79	0.15	0.55	6 of 6
AAAHHH_9	0.61	4.71	0.71	0.15	0.56	6 of 6
AAAHHH_7	0.59	4.71	0.67	0.14	0.55	6 of 6

AAAHHH_8	0.59	6.47	0.85	0.13	0.55	6 of 6
AAAHHH_6	0.59	4.12	0.7	0.14	0.55	6 of 6
AAAHHH_3	0.59	5.88	0.71	0.13	0.55	6 of 6
AAAHHH_4	0.58	4.71	0.71	0.13	0.55	6 of 6
AAAHHH_1	0.57	4.71	0.7	0.12	0.54	6 of 6
AAAHHH_5	0.53	4.71	0.67	0.1	0.54	6 of 6
AAAHRR_1	0.49	1.77	0.38	0.15	0.52	6 of 6

Table S2 - The table shows the pharmacophore hypotheses as well as their Phase hypo scores beside the BEDROC (Binary Ensemble Differential Relaxation Organizer) metric, which is used to evaluate the performance of pharmacophores and the EF1 (Enrichment Factor 1%), which is a statistical metric commonly used in virtual screening and drug discovery to evaluate the ability of a computational method to identify the most promising hits from a large database of compounds. The hypothesis that ranked highest based on such criteria was AAAHHH_2.

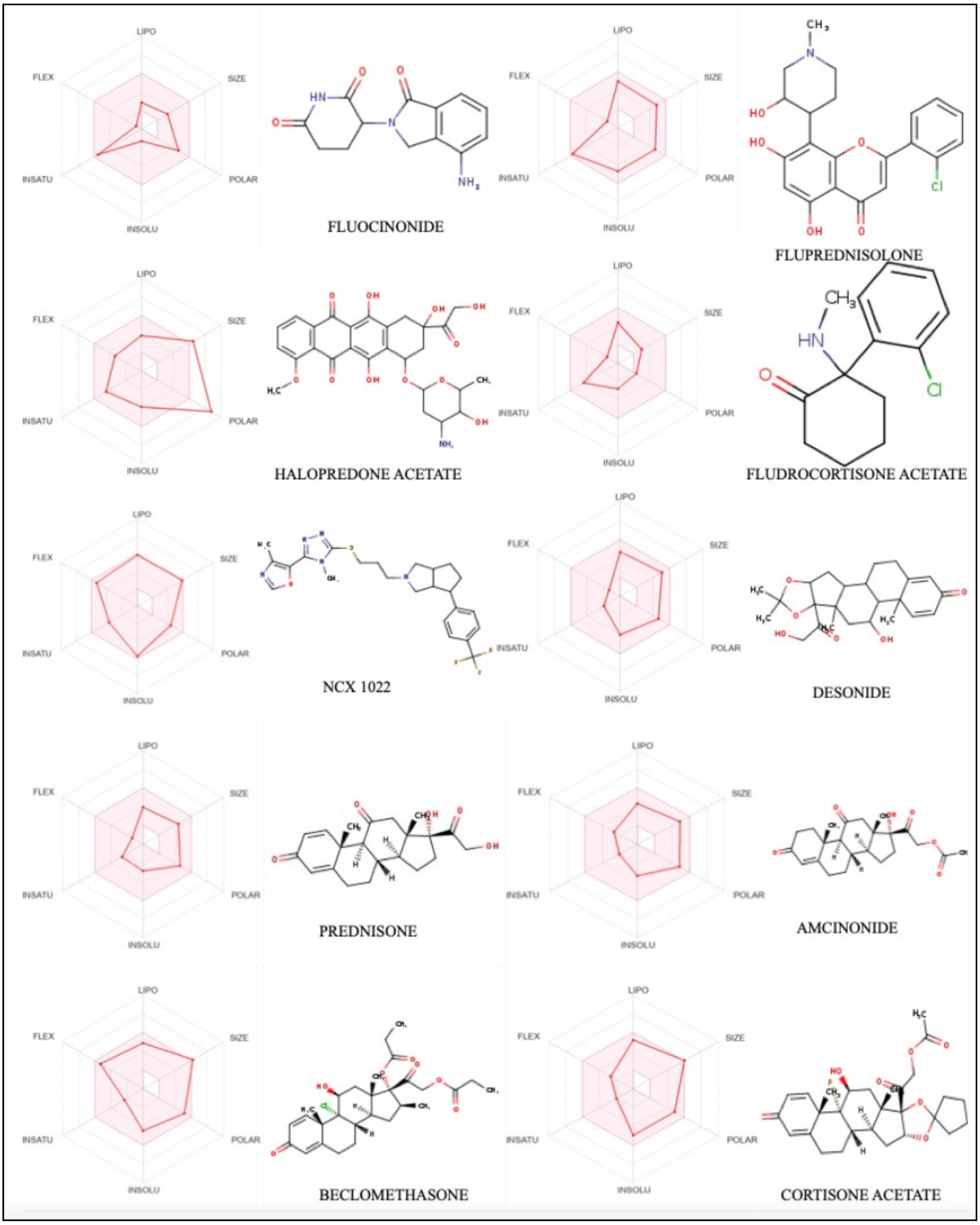

Figure S1: Physicochemical properties summarising ADME properties and 2D structures of the top ranked drugs resulting from the virtual screening. The red area represents ideal ADME values for pharmaceutical drugs. The ADME analysis reveals that NCX1022 shows higher metabolism rate compared with the other drugs. Moreover, NCX1022 shows medium oral absorption, while the other drugs from the top 10 showed high oral absorption. These findings show that the top 10 ranked drugs have medium to high ADME values, which is a good predictive factor for the bioavailability and for the maximum therapeutic efficacy.
